# On Vacciola, &c.

**Published:** 1805-01-01

**Authors:** 


### *To the Editors of the Medical and Physical Journal.*

GENTLEMEN,

MINE of the 3d of November, against the word VACCIOLA, I beg leave in some measure to retract; but, if the word was by me termed *a mere barbarism*, it was so on the ground of Dr. Walker's defence of its being *only* "for the sake of euphony, and because of similitude." (Medical and Physical Journal, No. lxix. p. 440.)

He should rather have defended it, as I now will, thus: Dr. STOKES, from *Vacca*, had a right to coin *Vaccia*, to express κατ' ἐξοχὴν, that particular bovine disorder, in which mankind is at present so greatly interested. But, as it is to the animal probably as *mild* an affection, as since its communication from the cow it has been to the human species, it may rather deserve the coinage of the diminutive *Vacciola*, to express a disease, which but in a small degree interrupts the health of either the brute or the human patient of it. Having established *Vacciola* upon this ground of preference to *Vaccia;* the at least equal euphony of the former, and also the similitude of its middle syllables to those of the long established Variol*æ*, may be adduced as additional arguments in its favor.

*Văcciŏlă*, on such grounds, may stand, as expressive of the mild bovine disease. But, it may be said, Why may not another medical authority coin *Vaccīna* or *Vaccīnŭlă?* I will answer, Vacciola is a substantive, coined *immediately* from a substantive or substantives; whereas Vaccina, a substantive, is coined from vacca *mediately* thro' the adjective vaccina. Vacciola is solely on that account preferable to Vaccina, without regard to the vaccine articles of human food.

But, Vaccia and Vacciola, the latter preferable perhaps even to good compounds, are coinages, and nothing more. They express only what it is agreed they shall express. Vaccævāra and Bōūnŏsŭs, legitimate compounds, and bearing a precise meaning, may, with my consent, readily yield to the coinage Vacciola of Dr. Stokes; who, rather than Dr. W. may be entitled to this apology from me. Vácciola, both accent and quantity upon the first syllable, which alone is long, is a pæon primus. Vărĭŏl*æ* is a pæon 4tus, accent on the i.

The compound, which Dr. W. (in note, p. 440, as before,) has requested, may be, ANDRONOSUS, a first pæon, or the first syllable only long. Thucydides, in his description of the plague at Athens, terms it, if I rightly recollect, Νοσος, κατ' ἐξοχὴν, because of its having been pre-eminently destructive. The same reason may support the term Andiŏnŏsŭs. Bounosus is that bovine disorder, which κατ' ἐξοχὴν, or pre-eminently, at present interests the world. The inoculation of it might also be termed, *Jēnnĕrĭă;* thence, Jenneriation, Jenneriator, to jenneriate, and jennerious matter.

Your's, &c.

